# Factors associated with the worsening of COVID-19 symptoms among cohorts in community- or home-isolation care in southern Thailand

**DOI:** 10.3389/fpubh.2024.1350304

**Published:** 2024-03-20

**Authors:** Thanit Sila, Wisanuwee Suriyaamorn, Chanavee Toh, Songyos Rajborirug, Smonrapat Surasombatpattana, Paramee Thongsuksai, Chanon Kongkamol, Sarunyou Chusri, Phoomjai Sornsenee, Prasit Wuthisuthimethawee, Raya Chaowanawong, Surasak Sangkhathat, Thammasin Ingviya

**Affiliations:** ^1^Department of Pathology, Faculty of Medicine, Prince of Songkla University, Songkhla, Thailand; ^2^Department of Health Science and Clinical Research, Faculty of Medicine, Prince of Songkla University, Songkhla, Thailand; ^3^Division of Digital Innovation and Data Analytics, Faculty of Medicine, Prince of Songkla University, Songkhla, Thailand; ^4^Department of Epidemiology, Faculty of Medicine, Prince of Songkla University, Songkhla, Thailand; ^5^Department of Family Medicine and Preventive Medicine, Faculty of Medicine, Prince of Songkla University, Songkhla, Thailand; ^6^Department of Internal Medicine, Prince of Songkla University, Hat Yai, Songkhla, Thailand; ^7^Faculty of Medicine, Department of Family Medicine and Preventive Medicine, Prince of Songkla University, Hat Yai, Songkla, Thailand; ^8^Department of Emergency Medicine, Faculty of Medicine, Prince of Songkla University, Hat Yai, Songkhla, Thailand; ^9^Faculty of Nursing, Prince of Songkla University, Hat Yai, Songkla, Thailand; ^10^Department of Biomedical Sciences and Biomedical Engineering, Faculty of Medicine, Prince of Songkla University, Songkhla, Thailand; ^11^Faculty of Medicine, Translational Medicine Research Center, Prince of Songkla University, Songkhla, Thailand

**Keywords:** COVID-19, vaccine, home-isolation, community-isolation, time-to-referral, Southern Thailand

## Abstract

**Introduction:**

This study aimed to investigate factors associated with time-to-referral due to worsening symptoms in patients with laboratory-confirmed COVID-19 in southern Thailand. While underlying diseases have been evaluated to assess COVID-19 severity, the influence of vaccinations and treatments is also crucial.

**Methods:**

A cohort of 8,638 patients quarantined in home or community isolation with laboratory-confirmed COVID-19 was analyzed. Survival analysis and the Cox proportional hazard ratio were employed to assess factors influencing time-toreferral.

**Results:**

Age ≥ 60 years, neurologic disorders, cardiovascular disease, and human immunodeficiency virus infection were identified as significant risk factors for severe COVID-19 referral. Patients who received full- or booster-dose vaccinations had a lower risk of experiencing severe symptoms compared to unvaccinated patients. Notably, individuals vaccinated during the Omicron-dominant period had a substantially lower time-to-referral than those unvaccinated during the Delta-dominant period. Moreover, patients vaccinated between 1 and 6 months prior to infection had a significantly lower risk of time-to-referral than the reference group.

**Discussion:**

These findings demonstrate early intervention in high-risk COVID-19 patients and the importance of vaccination efficacy to reduce symptom severity. The study provides valuable insights for guiding future epidemic management strategies and optimising patient care during infectious disease outbreaks.

## 1 Introduction

Severe Acute Respiratory Syndrome Coronavirus 2 (SARS-CoV-2), the causative agent of coronavirus disease (COVID-19), has spread rapidly worldwide since its emergence (1–3). The pandemic has caused significant morbidity and mortality globally and poses considerable challenges to healthcare systems, including patient overabundance (3–5). The World Health Organization (WHO) is at the forefront of providing management and prevention guidelines for COVID-19 (6). Although the disease can cause severe illness requiring hospitalization, a considerable proportion of patients experience asymptomatic/mild symptoms, who exhibit general symptoms of illness or symptoms like influenza, and can be managed in isolated facilities. Home isolation (HI) is a remote service with separate quarantine rooms which enable patients to self-care without infecting others. In cases without proper isolation spaces, community isolation (CI) is provided at hotels, temples, and colleges. However, self-care remains the primary mode of care in CI under the remote supervision of healthcare workers.

At the beginning of the COVID-19 pandemic in Thailand, all patients were being admitted to hospitals or field hospitals fully staffed with medical personnel. After the outbreak of the Delta (from June 2021 to January 2022) and omicron variants (from February 2022 to present) (7), thousands of individuals were infected daily (8–10). Hence, the Thai Ministry of Public Health teased WHO prevention measures by instituting hotel isolation, CI, and HI to prevent the over-occupancy of hospital beds, provided that patients have either mild or no symptoms (O_2_ saturation > 94%) (11–14), or no chronic disease which may aggravate later. The Thai Ministry of Public Health has classified patients which are prone to deterioration, called “608” group. The criteria of “608” included presence of older age (“60”) and/or seven underlying conditions, including obesity, cardiovascular disease, chronic respiratory disease, chronic kidney disease, brain disorders, diabetes, and cancer and pregnancy (“8”) (15). However, not all patients meeting criterion 608 under the care of Songklanagarind Hospital were promptly admitted. Patients without severe symptoms were quarantined under HI/CI, despite meeting the criteria that would require them to be hospitalized immediately and receive antiretroviral medications such as favipiravir. After being vaccinated against COVID-19, according to criterion 608, these patients could remain in HI or CI under the close supervision of medical staff if they were asymptomatic and met all other wellness criteria.

Despite numerous support measures, including daily surveillance and remote follow-up by medical teams using the latest technology, patients with HI and CI (10 days) require hospitalization owing to worsening symptoms (16–19). Previous studies have focused on various factors, particularly underlying diseases, and patient characteristics, for their potential associations with COVID-19 severity and mortality (20–27). However, certain factors such as the type and number of vaccinations and treatments influence the reduction in severity, thereby lowering the chances of hospital admission (27). Consequently, this observational study aimed to investigate the risk factors for hospital admission in patients with HI/CI by comparing time-to-referral as the primary outcome. Data are intended to be used in guideline development to deal with at-risk individuals in a timely manner and as a strategy for managing future epidemics.

## 2 Materials and methods

### 2.1 Study design and population

This prospective cohort study used multiple large datasets derived from the COVID-19 quarantine system managed by a university in southern Thailand. This study included patients with laboratory-confirmed COVID-19 from Songkhla Province who were registered for quarantine care at Songklanagarind Hospital, Prince of Songkla University, and personnel and their relatives who were quarantined in HI or CI provided by Songklanagarind Hospital, Hat Yai District, Songkhla Province, Thailand.

### 2.2 Inclusion and exclusion criteria

**Figure 1:**
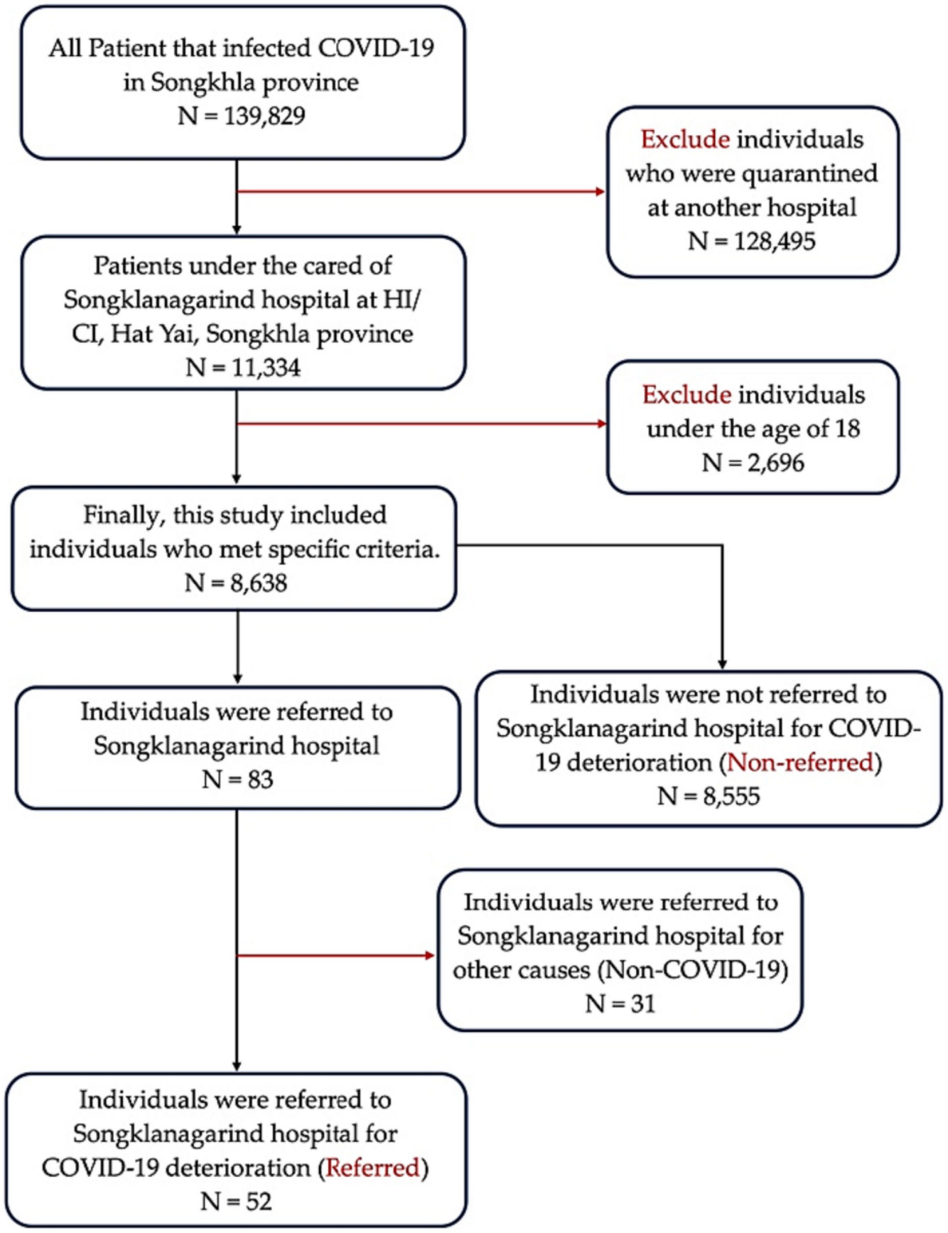
Schematic representation for the inclusion and exclusion criteria. HI, home isolation; CI, community isolation.

The inclusion criteria of the patients were as follows: (1) aged ≥18 years (2) under the care of HI/CI managed by Songklanagarind Hospital (Prince of Songkla University Hospital) from July 1, 2021, to July 31, 2022, and (3) confirmed positive for COVID-19. The exclusion criteria were as follows: (1) pregnant women; (2) patients who were initially admitted to the hospital; (3) inability to contact or provide less than half of the requested information; (4) referred to other quarantine states, altered the network of care later; (5) referrals not related to the increased severity of COVID-19; or (6) patients hospitalized for reasons other than worsening of the condition, such as to atteend to a referred child, appendicitis, or childbirth (Figure 1).

### 2.3 Definition and diagnostic criteria

Patients who tested positive for SARS-CoV-2 using an antigen test kit (ATK) or reverse transcription-polymerase chain reaction (RT-PCR) were considered positive for COVID-19. The first day of each COVID-19 episode (Day 0) was defined as the day when the patients were confirmed positive for COVID-19 by RT-PCR and/or ATK.

The age of each patient was calculated by deducting the date of birth from the time of the initial COVID-19-related occurrence.

The study used criteria-608 to assess the presence of any underlying diseases. This implied that patients with confirmed COVID-19 also had at least one of the following conditions: diabetes mellitus, cardiovascular disease, chronic respiratory disease, chronic kidney disease, cerebrovascular disease, obesity (defined as having a body mass index of ≥30.0 kg/m^2^) (28, 29), or cancer. Pregnancy prior to SARS-CoV-2 infection was also considered an underlying condition; however, it was not included in this study.

The severity spectrum of COVID-19 is as follows:

Asymptomatic: A person who has been confirmed to have COVID-19 through virological testing, either with real-time PCR, but does not have any external expressions or symptoms suggestive of COVID-19.

Mild symptom: fever, fatigue, anosmia, anorexia, headache, myalgia, diarrhea, loss of taste or smell, and upper respiratory infection symptoms such as cough, sore throat, and shortness of breath.

Moderate symptoms: This classification includes individuals experiencing increased breathing difficulty upon exertion, chest tightness or pain, difficulty breathing at rest, and persistent high-grade fever. These symptoms suggest a more advanced stage of COVID-19 requiring closer monitoring. This includes individuals with an oxygen saturation (SpO_2_) of 94% or higher who exhibit symptoms of the lower respiratory tract or have undergone clinical imaging of the lungs.

Severe symptoms: septic shock, acute respiratory distress syndrome, disseminated intravascular coagulation, macrophage activation syndrome, pneumonia. This includes individuals with a respiratory frequency > 30 breaths per minute, SpO_2_ < 94%, a ratio of arterial partial pressure of oxygen to fraction of inspired oxygen (PaO_2_/FiO_2_) < 300 mmHg, or lung infiltrates >50%.

The discharge date was disinvited by those who were admitted for the full 10-day follow-up period without an event and were administratively censored.

Time_0_ was the day the patient’s COVID-19 status was confirmed using RT-PCR or ATK. According to our database, 95% of the participants were confirmed to have COVID-19 on the first day after symptom onset. Only 5% of patients received positive COVID-19 results within 1–2 days of symptom onset. For these individuals, the day of symptom onset was considered as time_0_.

Time to referral (Time_t_), measured while the patients were in HI or CI, was the outcome of interest. At this point, physicians decided to refer the patient to the main hospital based on deteriorating symptoms.

To assess the effect of vaccine timing on the risk of COVID-19 infection, the time between the recent vaccination or last dose of immunization and the confirmation of COVID-19 infection was gathered, which was referred to as the “period of last vaccination.”

To identify various SARS-CoV-2 variants, we categorized the patients into two distinct time periods based on the dominant variants in the area. Patients who tested positive before February 1, 2022, were assigned to the Delta-dominant period, whereas those who tested positive thereafter were assigned to the Omicron-dominant period. The period of the dominant variant was determined by random specimen collection from patients with COVID-19 in Songkhla, Thailand, followed by next-generation sequencing analysis. All viral sequence data have been submitted to GISAID for global data sharing.^1^

### 2.4 Data source

We constructed a cohort of 8,638 patients in HI/CI with laboratory-confirmed SARS-CoV-2 infection. Clinical and epidemiological data were obtained from the enrolled patients who completed daily self-reporting questionnaires until they were discharged or referred for deteriorating symptoms. In the case of referral, electronic medical records or charts were maintained by physicians and nurses in the Songklanagarind Hospital database. Data on patient demographics, laboratory test results, medication administration, past and present diagnoses, clinical notes, and vaccination histories were collected. In addition, specific data on the most severe COVID-19 manifestations occurring during quarantine in HI/CI patients were retrospectively extracted from the questionnaires.

The study entry date was defined as the date on which the patient received hospital RT-PCR confirmation of COVID-19 positivity or self-examination using the ATK method. The index dates ranged from July 1, 2021 to July 31, 2022. The study endpoint was the time from the study index to either referral from HI/CI to the hospital or discharge. Recovered patients had their data censored on the date of discharge. The number of patients who were either referred, discharged, or were still admitted to the hospital on July 1, 2021, was recorded, and the duration of HI/CI was determined.

### 2.5 Target population

Patients in HI or those isolated in certain healthcare institutions were selected using the same inclusion and exclusion criteria as those used in our study. Furthermore, the Songkhla Provincial Public Health Ministry has comparable patient evaluation criteria, particularly those identifying 608 patients, with the primary goal of examining their clinical symptoms when infected with SARS-CoV-2.

### 2.6 Outcome

The referral criteria for this study included patients with confirmed COVID-19 infection using RT-PCR or ATK testing. Patients eligible for quarantine in extended hospital facilities under the care of Songklanagarind Hospital were referred if they exhibited symptoms of deteriorating health, necessitating subsequent hospitalization from HI/CI. The criteria for hospitalization or closer monitoring in HI and CI for COVID-19 patients include:

- Increased breathing difficulty during physical activities, chest tightness or pain, or trouble breathing while at rest.
- Persistent high-grade fever not alleviated by standard treatments.
- Any symptoms or conditions that necessitate close monitoring, such as abdominal pain to exclude surgical requirements or other serious complications.
- Oxygen saturation (SpO_2_) levels falling below 95%.

### 2.7 Statistical analyses

Patients’ baseline characteristics, including age, sex, presence of underlying diseases, type of vaccine, number of vaccine doses, duration of last vaccination, treatment, and dominant variant duration, were examined using descriptive statistics. Continuous data are presented using either the mean or median, depending on their distribution patterns. Categorical data are presented as proportions. Differences between the groups of variables were examined using the chi-squared test, and the resulting *p*-values were used to determine the statistical significance of these differences. The log-rank test was used to examine categorical data associations between the in-group of the variable and time-to-event. The probability of survival was presented using Kaplan–Meier curves. Statistical significance was set at a *p* < 0.05. For the type of vaccine, number of vaccine doses, and duration of the last vaccination, *p*-values were calculated using Peto-Peto (30), to address the crossover problem of the Kaplan–Meier curves.

Univariate and multivariate Cox proportional hazard models were constructed to investigate factors associated with time-to-event (time-to-referral). Initially, univariate Cox regression analyses were performed to examine the associations between each prospective predictor variable and time-to-referral. Hazard ratios (HRs) and the corresponding 95% confidence intervals were calculated. Independent associations between various factors and time-to-referral were assessed using a multivariable Cox regression model. The original multivariable model included all possible predictor variables (*p* < 0.20 in the univariate analysis). After considering potential confounding variables, the multivariate model provided an adjusted estimate of the association between each variable and time-to-referral. The final multivariable model, which was constructed using a stepwise backward selection process based on the Akaike Information Criterion (AIC) procedure (31, 32), was regarded as the best-fit model since it captured the most important variables associated with time-to-referral. To determine the strength and significance of the associations, the HRs, 95% confidence intervals, and *p*-values of the final model were represented using a forest plot.

Subgroup analysis of patients vaccinated with at least one type or dose of vaccine (excluding patients unvaccinated for COVID-19) was performed to explore the association between the period of last vaccination and time-to-referral using Cox proportional hazards ratio. The final multivariate model of the subgroup analysis was constructed using a stepwise backward selection process based on the AIC procedure and was regarded as the best-fit model. All analyses were performed using R (33).

### 2.8 Ethical statement

This study was approved by the Human Research Ethics Committee of the Faculty of Medicine of Prince Songkla University, Thailand (REC: 65-133-9-1). The researchers were permitted to retrieve clinical data and daily self-reported questionnaires from the hospital database with a renunciation of informed consent. All data were anonymized prior to analysis and implementation. The study was conducted in accordance with the principles of the Declaration of Helsinki, as confirmed by the researchers.

## 3 Results

### 3.1 Study design

**Figure 2:**
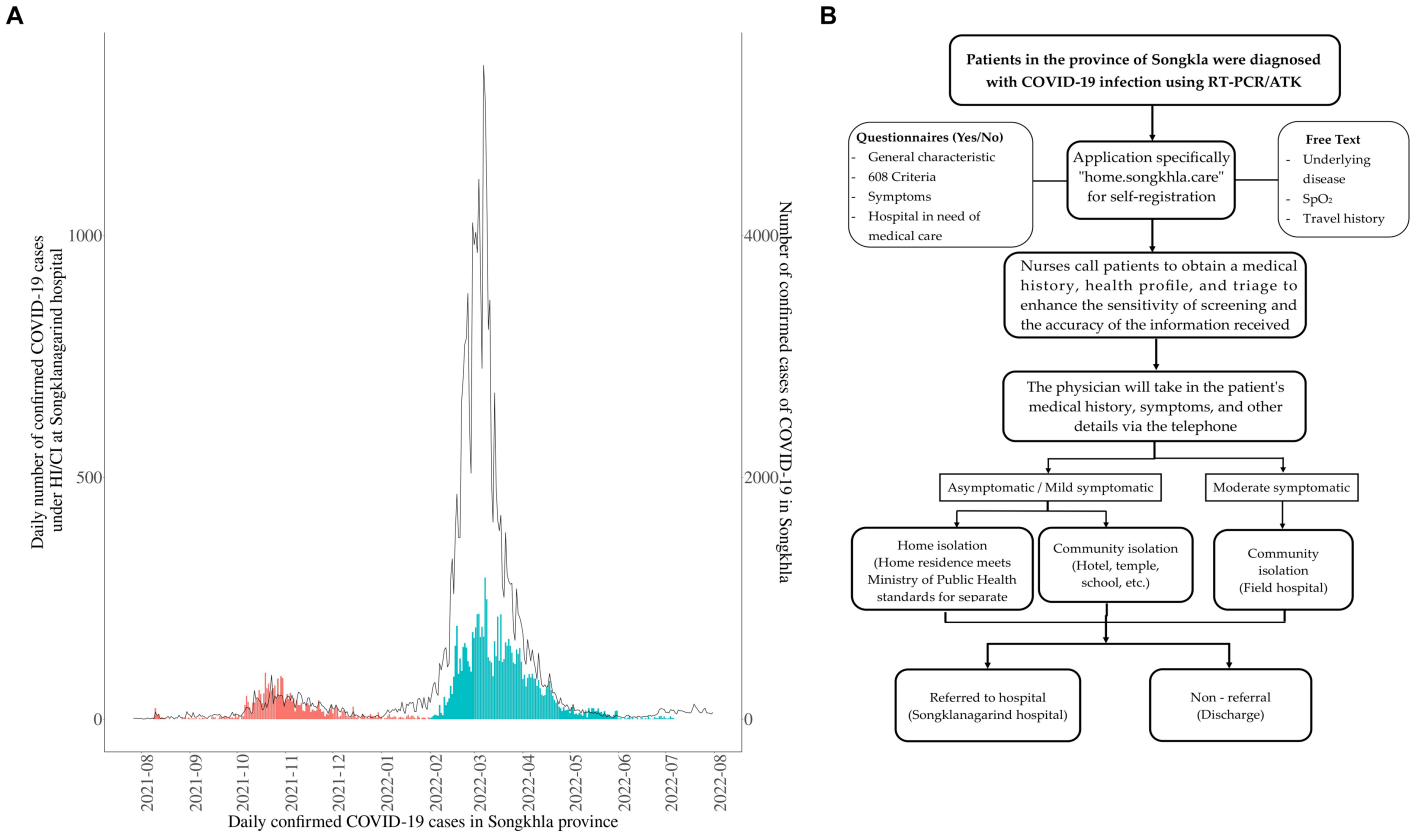
Overview of the 8,638 patients with coronavirus disease (COVID-19) under the care of HI/CI in this study. **(A)** Temporal distributions of confirmed COVID-19 cases in the Songkhla Province (line, right axis) and COVID-19 patients under home isolation (HI)/ community isolation (CI) care at the hospital (bars, left axis). The study period was divided into the periods of the Delta (orange) and omicron (blue) variants. Confirmed case data were obtained from The Ministry of Public Health of Thailand (34). **(B)** Schematic representation of the study design during HI/CI until recovery from COVID-19 or referral to the Songklanagarind Hospital for further admission.

In the final cohort, we included 8,638 patients with laboratory-confirmed SARS-CoV-2 infection who were under HI/CI care at the hospital from July 2021 to July 2022. The study participants were screened by obtaining medical histories through nurses by completing application-based inquiries (namely “Songkhla care”) and the retrieval of a database containing previous medical records from institutions. Further, the cohort was examined by a physician and the symptoms were reevaluated, to determine the quarantine location (HI, CI, or hospitalization). Subsequently, medical staff communicated daily to inquire about vital signs until the patient recovered or was hospitalized due to disease deterioration (Figure 2A). During the study period, there were outbreaks of two variants [Delta-dominant period (orange) and Omicron-dominant period (blue); Figure 2A].

### 3.2 Demographic characteristics and underlying diseases of COVID-19 patients

Majority of the patients with COVID-19 were female (62.1%). Patients aged >60 years comprised a relatively modest percentage (13.2%, median age, 67 years) of all patients with COVID-19 in the care of Songklanagarind Hospital. Diabetes mellitus, obesity, chronic lung disease, neurological disorders, cardiovascular disease, chronic kidney disease, chronic liver disease, immunocompromised diseases, cancer, depression, and systemic lupus erythematosus (SLE) were the most common underlying diseases (Table 1). However, cancer, depression, and SLE were not reported among the patients with COVID-19 who were referred to the hospital at the end of this study. In the log-rank test, sex, age, diabetes mellitus, hypertension, neurological disorders, chronic kidney disease, cardiovascular disease, and human immunodeficiency virus (HIV) infection were significantly associated with time-to-referral.

**Table 1:** Characteristic and underlying diseases of patients with COVID-19.

Variables	Category	Total *N* = 8,638 (%)	Non-referred *N* = 8,586 (%)	Referred *N* = 52 (%)	*P*-value
Sex					
	Female	5,362 (62.07)	5,340 (99.59)	22 (0.41)	0.005
	Male	3,276 (37.93)	3,246 (99.08)	30 (0.92)	
Age (years)					
	18–59	7,497 (86.79)	7,466 (99.59)	31 (0.41)	< 0.001
	60 and older	1,141 (13.21)	1,120 (98.16)	21 (1.84)	
Diabetes mellitus					
	No	8,217 (95.13)	8,176 (99.50)	41 (0.50)	< 0.001
	Yes	421 (4.87)	410 (99.87)	11 (0.13)	
Hypertension					
	No	7,966 (92.22)	7,926 (99.50)	40 (0.50)	< 0.001
	Yes	672 (7.78)	660 (98.21)	12 (1.79)	
Obesity					
	No	7,554 (87.45)	7,511 (99.43)	43 (0.57)	0.138
	Yes	1,084 (12.55)	1,075 (99.17)	9 (0.83)	
Chronic lung disease					
	No	7,957 (92.12)	7,909 (99.40)	48 (0.60)	0.990
	Yes	681 (7.88)	677 (94.41)	4 (0.59)	
Neurologic disorders					
	No	8,577 (99.29)	8,535 (99.51)	42 (0.49)	< 0.001
	Yes	61 (0.71)	51 (83.61)	10 (16.39)	
Chronic kidney disease					
	No	8,587 (99.41)	8,538 (99.43)	49 (0.57)	< 0.001
	Yes	51 (0.59)	48 (94.12)	3 (5.88)	
Cardiovascular disease					
	No	8,471 (98.10)	8,432 (99.54)	39 (0.46)	< 0.001
	Yes	167 (1.90)	154 (92.22)	13 (7.78)	
Chronic liver disease					
	No	8,606 (99.63)	8,555 (99.41)	51 (0.59)	0.482
	Yes	32 (0.37)	31 (96.87)	1 (3.13)	
HIV infection					
	No	8,588 (99.42)	8,538 (99.42)	50 (0.58)	0.028
	Yes	50 (0.58)	48 (96.00)	2 (4.00)	

HIV, Human Immunodeficiency Virus.

Two primary reasons for referral were as follows: (1) COVID-19-related severe symptoms, including respiratory complications and/or SpO_2_ < 94% (*n* = 39), confirmed pneumonia (*n* = 13), or headache with fever >39°C and chest pain (*n* = 4) (Supplementary Table S1), (2) other referral causes (not outcomes for this study), such as accident, appendicitis, or hospitalization of mother with breast-feeding infant.

### 3.3 Vaccination, treatment, and variant period of patients with COVID-19

Majority of the patients (68.70%) were vaccinated prior to testing positive for COVID-19 (68.70%) and were categorized as (1) non-mRNA [26.40% using inactivated virus vaccine (Sinovac, Sinopharm) and viral vector vaccine (AstraZeneca)], (2) mRNA (8.60% using Pfizer and Moderna vaccines), and (3) combined vaccine (33.80%, non-mRNA combined with mRNA vaccine). There was no significant difference in the rate of referral between the vaccine types. However, the ratio of referrals was significantly different among vaccination doses, with the lowest ratio in patients who received full or booster doses. A small percentage of patients received favipiravir (2.46%) since their condition met their physicians’ risk criteria, including older adults and those with underlying medical conditions. Moreover, a large proportion of patients (78.64%) were diagnosed in the omicron-dominant outbreak and the referral rates were significantly different between the Omicron and Delta periods. Additionally, all variables were tested for their association with time-to-referral using the log-rank test, and the findings were similar to the chi-square results (Table 2). The data are displayed as Kaplan–Meier plots (Supplementary Figure S1).

**Table 2:** Vaccination status, treatment, and period of dominant COVID-19 variant.

Variables	Detail	Total *N* = 8,638 (%)	Non-referred *N* = 8,586 (%)	Referred *N* = 52 (%)	*P*-value
Vaccine type	Non-vaccinated	2,702 (31.28)	2,679 (99.15)	23 (0.85)	0.104
	Non-mRNA	2,278 (26.37)	2,264 (99.39)	14 (0.61)	
	mRNA	739 (8.62)	734 (99.32)	5 (0.68)	
	Combination	2,919 (33.88)	2,909 (99.65)	10 (0.34)	
Number of vaccine doses	Non-vaccinated	2,702 (31.28)	2,679 (99.15)	23 (0.85)	0.001
	Partial dose	415 (4.80)	408 (98.31)	7 (1.69)	
	Full or booster	5,521 (63.92)	5,499 (99.60)	22 (0.40)	
Treatment	Non-favipiravir	8,434 (97.64)	8,387 (99.44)	47 (0.56)	0.003
	Favipiravir	204 (2.46)	199 (98.55)	5 (2.45)	
Period of dominant variant	Delta	1,845 (21.36)	1,824 (99.86)	21 (1.14)	0.001
	Omicron	6,793 (78.64)	6,762 (99.54)	31 (0.86)	
Duration of last vaccination	Non-vaccinated	2,707 (31.34)	2,683 (99.11)	24 (0.89)	0.008
	Less than 1 month	829 (9.60)	820 (98.91)	9 (1.09)	
	1–6 months	4,604 (53.30)	4,588 (99.65)	16 (0.35)	
	More than 6 months	498 (5.86)	495 (94.40)	3 (0.60)	

### 3.4 Factors associated with time-to-referral due to deterioration of symptoms

**Figure 3:**
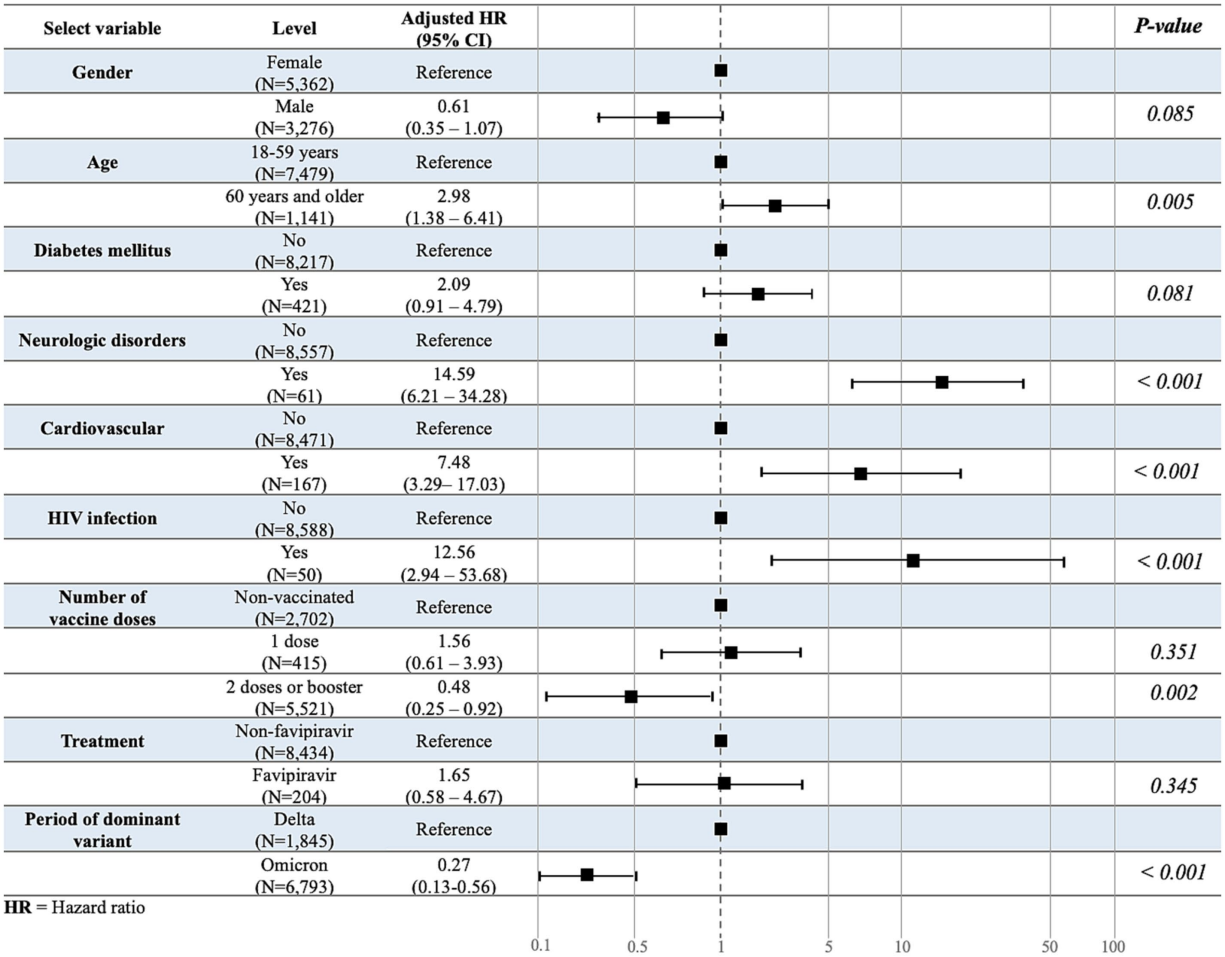
Multivariate association study for the time-to-referral. Multivariate analysis was used to evaluate the variables associated with the outcome variable, time-to-referral.

Among the patients with COVID-19 quarantined in HI/CI, 0.6% were referred to the hospital after developing severe symptoms. In multivariable analyses, the risk of referral was higher among those aged ≥60 years than those aged 18–59 years (HR: 2.98; 95% confidence interval: 1.38–6.41; *p* = 0.005; Figure 2). Sex was not significantly associated with the number of referrals. With regard to underlying diseases, there was a significant risk of referral due to worsening of symptoms among patients with COVID-19 and a neurological disorder, cardiovascular disease, or HIV infection. Other underlying diseases were not significantly associated with an increased referral risk. In addition, patients immunized with at least two COVID-19 vaccine doses or booster doses were significantly less likely to experience severe symptoms necessitating referral, whereas the vaccine type and administration of favipiravir drug showed no association. Furthermore, those infected in the omicron-dominant period had a significantly lower risk of referral than those infected in the Delta-dominant period (Figure 3).

### 3.5 Elucidating the association between the vaccine type and the period of dominant virus variant on time-to-referral in patients with COVID-19

In our multivariable Cox regression model, which accounted for other covariates such as age, sex, underlying disease, and other factors involved in risk reduction, the individual effects of vaccine type were not statistically significant predictors of time-to-referral, whereas the period of the dominant variant was significant. However, when examining the interaction term between vaccine type and period of variation, we found a significant association between the combined vaccine and risk reduction during the Omicron-dominant period. Specifically, the combination vaccine, which was administered to individuals infected within the period of the Omicron variant was associated with a significantly lower risk of time-to-referral (HR = 0.13; 95% confidence interval: 0.02–0.78; *p* = 0.025; Table 3).

**Table 3:** Interaction between vaccine types and period of dominant variant on the risk of referral.

Variable	Level	Hazard ratio (95% CI)	*P*-value
Type of vaccine	Non-vaccinated	Reference	–
	Non-mRNA	0.40 (0.12–1.34)	0.140
	mRNA	0.16 (0.01–1.63)	0.121
	Combination	3.06 (0.64–14.61)	0.161
Variant	Delta	Reference	–
	Omicron	0.28 (0.11–0.72)	0.008
Interaction covariate			
Type of vaccine: Variant	Non-mRNA: Omicron	1.12 (0.25–4.97)	0.885
	mRNA: Omicron	3.27 (0.27–39.47)	0.351
	Combination: Omicron	0.13 (0.02–0.78)	0.025

### 3.6 Cox regression analysis evaluating the association of period of vaccination with time-to- referral among subgroups of patients who received the vaccine against COVID-19

Additionally, we conducted a subgroup analysis by excluding unvaccinated patients (Supplementary Table S2) and focused solely on those who received non-mRNA, mRNA, or combination vaccines (Supplementary Table S3). Among these subgroups, we investigated the impact of the period between the last vaccination and the occurrence of COVID-19 on outcome measures, specifically the time-to-referral.

**Figure 4:**
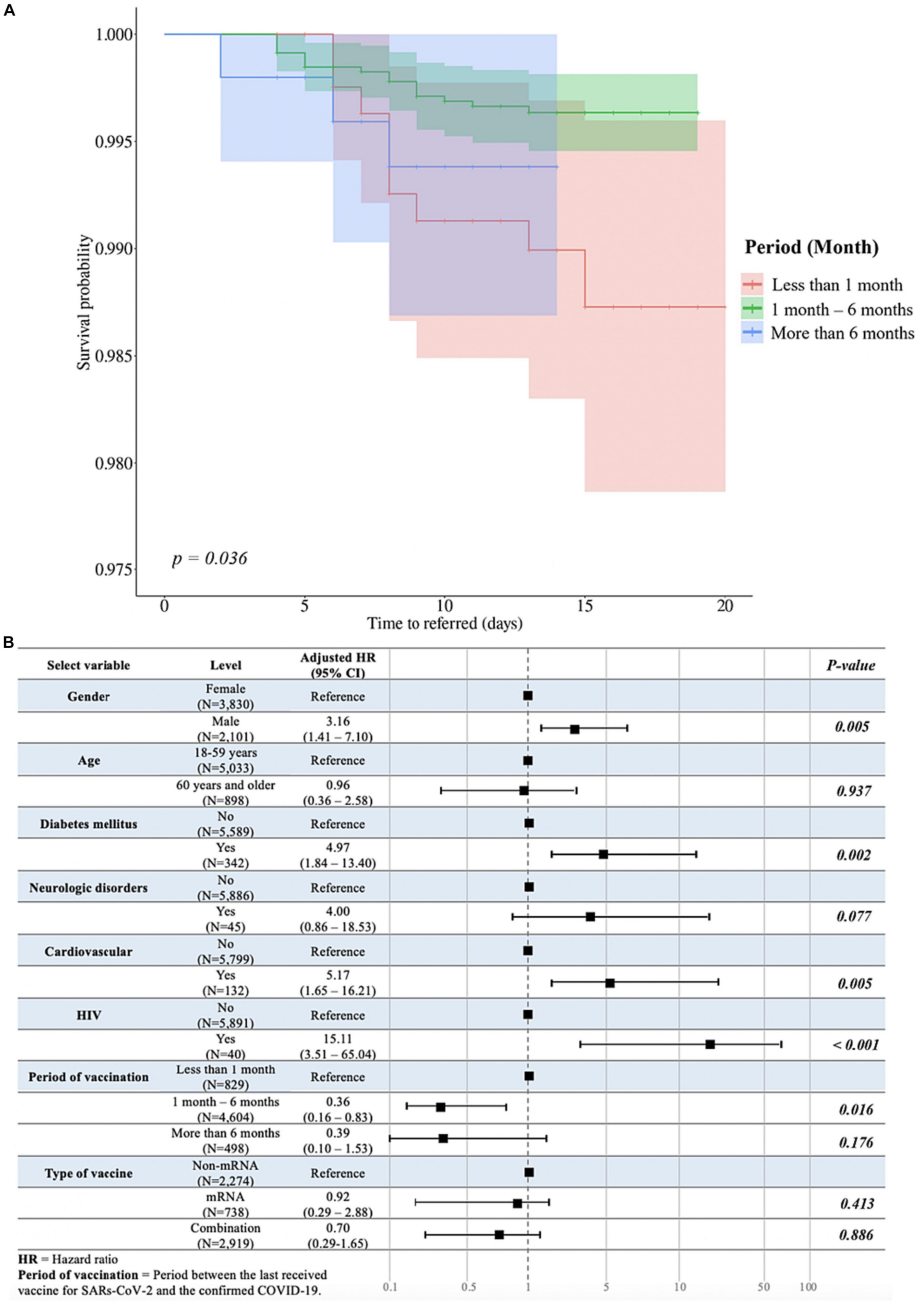
Analysis of duration between the last vaccination and the occurrence of COVID-19 and multivariable associations with time-to-referral, excluding unvaccinated participants. **(A)** The Peto-Peto test was used to examine the relationship between the duration after the last vaccination and the occurrence of COVID-19, specifically in relation to the time-to-referral period, and statistically significant differences were expressed as *p*-values. **(B)** Multivariate analysis evaluating the variables associated with the outcome variable and time-to-referral. Sex, age, presence of diabetes mellitus, neurological problems, HIV infection, the duration between the last vaccination and the occurrence of COVID-19, and the type of vaccine received were among the variables in the model. The adjusted hazard ratios and accompanying 95% confidence intervals for each variable are shown in the table, associating each variable to the time-to-referral.

Interestingly, the log-rank test revealed a significant difference in time-to-referral across different periods from the last vaccination (*p* = 0.03; Figure 4A). Furthermore, we found a significant association between the period from the last vaccination and the time-to-referral. Compared to the reference group, patients who were vaccinated 1–6 months prior to SARS-CoV-2 infection exhibited a significantly lower risk of experiencing a shorter time-to-referral (HR = 0.36; 95% CI = 0.16–0.83; *p* = 0.016; Figure 4B). In contrast, the time-to-referral was not significantly different between individuals vaccinated less than 1 month or more than 6 months before the infection compared to those of the reference group.

## 4 Discussion

This study aimed to predict the factors associated with the worsening of COVID-19 symptoms, leading to hospitalizations due to COVID-19. This study began during the fourth-wave outbreak in Thailand, characterized by the initial dominance of the Delta variant. Moreover, this variant has been reported to have the worst clinical outcomes worldwide (35). Later, Omicron outbreaks dramatically increased the number of infected individuals, but there were fewer severe cases and mortalities than those in the Delta-dominant period (7, 36). The patient in this study was quarantined for 10 days after the detection of SARS-CoV-2 on the first date, similar to previous a systematic review that revealed several Asian, European, and American nations with home-isolation policies (37). In addition, the medical team conducts daily follow-up calls and questionnaires to monitor patient symptoms, including the occurrence of unexpected symptoms and the impact of physical (38), mental, and emotional quarantine (39–41). Furthermore, this study was strengthened by the large number and variety of Songkhla patients who underwent HI and CI in primary care.

In this investigation, we utilized survival analysis, specifically Cox proportional hazard regression, to examine the effects of various associated factors on the deterioration of patients which were indicated by the referral to hospital. Multivariate analysis revealed that older age was associated with an increased risk of referral, similar to previous studies in which older age has been associated with high severity or mortality, independent of comorbidities (20, 26, 27, 42–44). Age-related upregulation in the expression of angiotensin-converting enzyme-2 (ACE-2), a SARS-CoV-2 spike protein receptor, hastens viral dissemination among older adults. In contrast, immunological dysregulation and alterations in the gut microbiota caused by aging may also contribute to the cytokine storm, a key marker of disease severity. Age-related disease severity is significantly influenced by changes in the levels of growth hormones and sex steroids, particularly in women, both of which play critical roles in immunological control. However, the outcome was not significantly different between males and females in our study, as observed previously (45). Moreover, our cohort had a higher proportion of females (62.1%), in contrast to many previous studies involving fewer female than male patients (44, 46, 47). Consequently, the probability of sex-based influences can have varying effects on the associated factors and outcomes in different settings (48); a previous study indicated that males are more susceptible to mortality or severity due to the significantly higher expression of the ACE-2 receptor in males than that in females (49).

In our cohort, some underlying diseases were significantly associated with an increased risk of symptom worsening. Specifically, patients with cardiovascular diseases, neurological disorders, and HIV infection were more likely to experience severe symptoms necessitating hospital referrals. Similar to previous studies, we found that cardiovascular comorbidities such as heart failure, acute myocardial injury, arrhythmias, cardiogenic shock, and coronary artery disease were associated with the progressive worsening of symptoms (50–54). Generally, ACE-2 expression is abundant in the heart and lungs and has been identified as a functional receptor (53, 55, 56). The virus decreases ACE-2 expression, which leads to an increase in angiotensin II, and ultimately, an increase in pulmonary vascular permeability, resulting in pulmonary edema (57). Additionally, antiviral drugs administered to patients with cardiovascular disease can increase the risk of symptom worsening (58). However, this study did not establish an association between antiviral drugs and cardiac disease nor did it identify additional risk factors. Neurological comorbidities are a significant determinant of COVID-19 severity, warranting a thorough evaluation from the earliest phases of infection (59, 60); studies on COVID-19 have revealed that approximately one-third of patients experience neurological symptoms (59, 61–63). Previous research on cerebrospinal fluid (CSF) and brain tissue indicates that SARS-CoV-2 infection causes immunological activation and inflammation inside the central nervous system (64) which corroborates prior observations in the CSF from living patients and is consistent with the discovery of SARS-CoV-2 nucleic acid or viral protein in the brains of those who succumbed to acute COVID-19 (65, 66). ACE-2 is overexpressed in specific regions of the human brain, including the substantia nigra and brain ventricles, as well as excitatory and inhibitory neurons in the middle temporal gyrus and posterior cingulate cortex which are target regions of SARS-CoV-2 (67). COVID-19 is substantially associated with severity or mortality in patients with HIV, as seen in cohorts of South Africa (ARR 2.14; 95% confidence interval: 1.70–2.70) (68), United Kingdom (AHR 2.59; 95% confidence interval: 1.74–3.84) (69), United States (AOR 1.29; 95% confidence interval: 1.16–1.44) (70) and New York (ARR 1.23; 95% confidence interval: 1.07–1.40) (71). Additionally, patients with HIV generally have decreased immunological and CD4 counts since the cytokine storm is one of the main mechanisms causing COVID-19-related morbidity and mortality (72). Therefore, in patients with HIV, significant immunosuppression and low CD4 levels may increase the risk of developing lymphopenia and the severity of COVID-19 (73). Thus, the results of this and previous studies support the idea that older individuals, including those with cardiovascular disease, neurological disorders, and HIV, should be closely monitored in the case of future epidemics.

Regarding COVID-19 vaccination, a significantly reduced risk of experiencing worsening symptoms was discovered in patients who received a full dose of the vaccine or a booster dose, compared to those in previous studies (74, 75). However, no association between the type of vaccine and patients’ referral was found in our study. The Omicron-dominant period had a lower AHR (0.28) than that in the Delta-dominant period, indicating a lower risk of symptom aggravation (76, 77). The high transformability and rising infection rate of the new variant may be risky; however, no adverse effects or additional fatalities were reported (78). After considering the interaction between the period of infection and type of vaccination, we observed that the interaction was strongly associated with a reduced risk of outcome. Although vaccine efficacy is significantly lower in the Omicron variant than that in the Delta variant (79–81), the use of heterologous vaccines in the first and second doses is associated with higher cellular and humoral immune responses and higher neutralizing antibody levels against COVID-19 than those with homologous vaccines (82, 83). A heterologous booster dose for people with complete primary vaccination scheduled with CoronaVac provided a high level of protection against COVID-19, including severe disease and death (84, 85). Similar results were observed with ChAdOx1 nCoV-19 vaccine as the first dose and mRNA vaccine (BNT162b2 or mRNA-1273) as the second dose (86, 87). Thus, the efficacy of the COVID-19 vaccine may depend on the variant mutation rather than the strain, and that the interaction between vaccine type and variant is important when assessing the effectiveness of vaccination strategies.

Our subgroup analysis also showed that among patients who received at least one dose of the COVID-19 vaccine, the duration between vaccination and COVID-19 exposure at 1–6 months was significantly associated with a reduction in the worsening of symptoms and referrals. Moreover, adenoviral vector vaccines have shown the highest efficacy after 3 weeks of vaccination (88). Similarly, in Thailand, a study reported that 97.26% of the participants who received the first dose of the AstraZeneca vaccine and 99.49% of those who received two doses of the Sinovac vaccine produce high levels of anti-COVID-19 antibodies after 4 weeks (89). In addition, according to a recent prospective cohort study in Scotland, the first dose of the Pfizer-BioNTech vaccine had an efficacy of approximately 91% against COVID-19 on hospital admission 28–34 days after immunization. The effect of the Oxford-AstraZeneca vaccine at the same time point was 88% (95% confidence interval, 75–94) (90, 91). However, previous studies on mRNA vaccines have demonstrated effective COVID-19 protection for only up to 6 months, with protection waning over the next 6 months (92, 93). However, to our knowledge, no study has definitively ascertained the highest immune activation time, and vaccine efficacies may change with regional variations. Few studies have investigated the efficacy and persistence of these vaccines.

This study had several limitations. The study period covered waves of epidemics of the Delta and Omicron variants (such as B.1.1.529, BA.1, and BA.2), which were inferred using the period or wave of the sub-lineage. Based on our previous study, the epidemiology was classified into a patient category. Many patients consumed *Andrographis paniculata* (Fah-Ta-Lai-Jone) without informing healthcare providers, preventing us from conclusively identifying an association between treatment and outcomes. In addition, our study did not categorize vaccine brands by manufacturer, but rather by type. Moreover, in Songkhla, fewer individuals received a single dose of the vaccine for the Omicron variant than for the Delta variant, and the period of last vaccination was collinear with the variant. Therefore, the variant variable was not used to correlate the period of recent vaccination with outcomes. If the patient’s condition worsened to the point of mortality or if they had severe respiratory symptoms and were unable to breathe independently, the patients were referred to the doctor’s discretion. This may be one of the reasons why the outcomes of this study were poor despite the diversity of patient backgrounds, availability of treatment, and large population size. Finally, a limitation of this study is that majority of patients with diabetes mellitus were well-controlled and regularly monitored, which may not fully represent the broader population of patients with COVID-19 and diabetes, who may have less optimal control or more severe disease outcomes.

In conclusion, this study aimed to provide information for medical personnel caring for patients, manage the overwhelming number of nursing patients with emerging diseases related to the respiratory tract, and identify the risk factors that influence the disease severity of a patient. Underlying diseases such as heart disease, neurological disorders, and HIV infection are the primary focus groups for monitoring and follow-up. Based on the present study findings, researchers recommend that high-risk patients be admitted to the hospital at the onset of infection. If this is not feasible, they should always be quarantined by caregivers. Additionally, old age is an influential risk factor for disease severity; however, not all older adults are at risk of developing severe diseases. Therefore, it is also necessary to consider the underlying diseases. Factors, such as vaccines may merely prevent disease severity, which is dependent on the outbreak of each strain. Therefore, further studies are warranted in this regard.

## Data availability statement

The original contributions presented in the study are included in the article/Supplementary material, further inquiries can be directed to the corresponding authors.

## Ethics statement

The studies involving humans were approved by the Institutional Review Board of the Faculty of Medicine, Prince of Songkla University, Thailand (protocol code REC: 65-133-9-1). The studies were conducted in accordance with the local legislation and institutional requirements. Written informed consent for participation was not required from the participants or the participants’ legal guardians/next of kin in accordance with the national legislation and institutional requirements. Written informed consent was obtained from the individual(s) for the publication of any potentially identifiable images or data included in this article.

## Author contributions

TS: Writing – original draft, Writing – review & editing. WS: Writing – review & editing. CT: Writing – review & editing. SR: Writing – review & editing. SSu: Writing – review & editing. PT: Writing – review & editing. CK: Writing – review & editing. SC: Writing – review & editing, Project administration, Resources. PW: Writing – review & editing, Methodology, Project administration, Resources. PS: Writing – review & editing, Project administration, Resources. RC: Writing – review & editing, Project administration, Resources. SSa: Writing – review & editing. TI: Writing – original draft, Writing – review & editing.

## Footnotes

^1^https://gisaid.org
