# Supplementary material for: Factors associated with the worsening of COVID-19 symptoms among cohorts in community- or home-isolation care in southern Thailand
Source: Front Public Health. 2024 Mar 20;12:1350304. doi: 10.3389/fpubh.2024.1350304 (PMC10987961; doi:10.3389/fpubh.2024.1350304)
Supplement: Supplementary file 1 [file Table_1.DOCX]

**Supplementary Table 1 Justifications for patient referral from HI/CI quarantine when symptoms worsen due to COVID-19**

| Vital sign | Sub vital sign | Referral (n = 52) | Total |
| --- | --- | --- | --- |
| 1. Respiratory system complications and/or SpO_2_ < 94% |  |  | 39 |
|  | 1.1 as pneumonia | 13 |  |
|  | 1.2 non-pneumonia | 26 |  |
| 1. Headache with a fever of more than 39 degrees |  |  | 9 |
|  | 2.1 Coughing and sputum that is bloody | 3 |  |
|  | 2.2 Nausea and vomiting | 3 |  |
| 1. Chest aches |  | 4 | 4 |

**Supplementary Table 2 Factors of subgroup analysis for the patient who received vaccine against COVID-19 at least one dose.**

| Factors | Detail | Total  N = 5,931 (%) | Non-referred  N = 5,903 (%) | Referred  N = 28 (%) | P-value |
| --- | --- | --- | --- | --- | --- |
| Gender | Female | 3,830 (64.58) | 3,821 (99.77) | 9 (0.33) | 0.001 |
|  | Male | 2,101 (35.42) | 2,082 (99.10) | 19 (0.90) |  |
| Age (years) | 18–59 | 5,033 (84.86) | 5,013 (99.60) | 20 (0.40) | 0.085 |
|  | ≥60 | 898 (15.14) | 890 (99.11) | 8 (0.89) |  |
| Diabetes mellitus | No | 5,589 (94.23) | 5,569 (99.64) | 20 (0.36) | <0.001 |
|  | Yes | 343 (5.77) | 334 (97.77) | 8 (2.33) |  |
| Hypertension | No | 5,387 (90.83) | 5,364 (99.44) | 23 (0.56) | 0.205 |
|  | Yes | 544 (9.17) | 539 (99.08) | 5 (0.92) |  |
| Obesity | No | 2,378 (40.09) | 2,371 (99.70) | 7 (0.30) | 0.150 |
|  | Yes | 3,553 (59.91) | 3,532 (99.40) | 21 (0.60) |  |
| Chronic lung disease | No | 5,419 (91.37) | 5,394 (99.54) | 25 (0.46) | 0.955 |
|  | Yes | 512 (8.63) | 509 (99.41) | 3 (0.59) |  |
| Neurologic disorders | No | 5,886 (99.24) | 5,860 (99.56) | 26 (0.44) | 0.005 |
|  | Yes | 45 (0.76) | 43 (99.56) | 2 (0.44) |  |
| Chronic Kidney disease | No | 5,894 (99.39) | 5,869 (99.58) | 25 (0.42) | <0.001 |
|  | Yes | 37 (0.61) | 34 (91.89) | 3 (9.11) |  |
| Cardiovascular disease | No | 5,799 (97.77) | 5,776 (99.60) | 23 (0.40) | <0.001 |
|  | Yes | 132 (2.23) | 127 (96.97) | 5 (3.03) |  |
| Chronic Liver disease | No | 5,904 (99.55) | 5,877 (99.54) | 27 (0.46) | 0.294 |
|  | Yes | 27 (0.45) | 26 (96.29) | 1 (3.71) |  |
| HIV disease | No | 5,891 (99.33) | 5,865 (99.56) | 26 (0.44) | 0.002 |
|  | Yes | 40 ()0.67 | 38 (95.00) | 2 (5.00) |  |
| Vaccine type | Non-mRNA | 2,274 (38.34) | 2,260 (99.39) | 14 (0.61) | 0.347 |
|  | mRNA | 739 (12.46) | 734 (99.32) | 5 (0.68) |  |
|  | Combination | 2,919 (49.22) | 2,909 (99.65) | 10 (0.34) |  |
| Number of  vaccine doses | Partial dose | 415 (7.00) | 408 (98.31) | 7 (1.69) | 0.001 |
|  | Full or booster | 5,521 (93.00) | 5,499 (99.60) | 22 (0.40) |  |
| Treatment | Non-favipiravir | 5,434 (91.62) | 5,915 (99.73) | 27 (0.27) | 0.121 |
|  | Favipiravir | 16 (8.38) | 15 (93.75) | 1 (6.25) |  |
| Period of dominant variant | Delta | 1,045 (17.62) | 1,034 (98.95) | 11 (1.05) | 0.006 |
|  | Omicron | 4,886 (82.38) | 4,869 (99.65) | 17 (0.35) |  |
| Duration of last vaccination | <1 month | 829 (13.98) | 820 (98.91) | 9 (1.09) | 0.008 |
|  | 1–6 months | 4,604 (77.63) | 4,588 (99.65) | 16 (0.35) |  |
|  | >6 months | 498 (8.39) | 495 (94.40) | 3 (0.60) |  |

**Supplementary Table 3 Pattern of combination vaccinated against COVID-19.**

| **Pattern** | **Dose 1** | **Dose 2** | **Dose 3** | **Dose 4** | **Number of patients received combination vaccines** |
| --- | --- | --- | --- | --- | --- |
| 1 | non-mRNA | non-mRNA | mRNA |  | 1,955 |
| 2 | non-mRNA | non-mRNA | mRNA | mRNA | 580 |
| 3 | non-mRNA | mRNA |  |  | 83 |
| 4 | non-mRNA | mRNA | mRNA |  | 31 |
