# Supplementary figures and images for: Factors associated with the worsening of COVID-19 symptoms among cohorts in community- or home-isolation care in southern Thailand
Source: Front Public Health. 2024 Mar 20;12:1350304. doi: 10.3389/fpubh.2024.1350304 (PMC10987961; doi:10.3389/fpubh.2024.1350304)

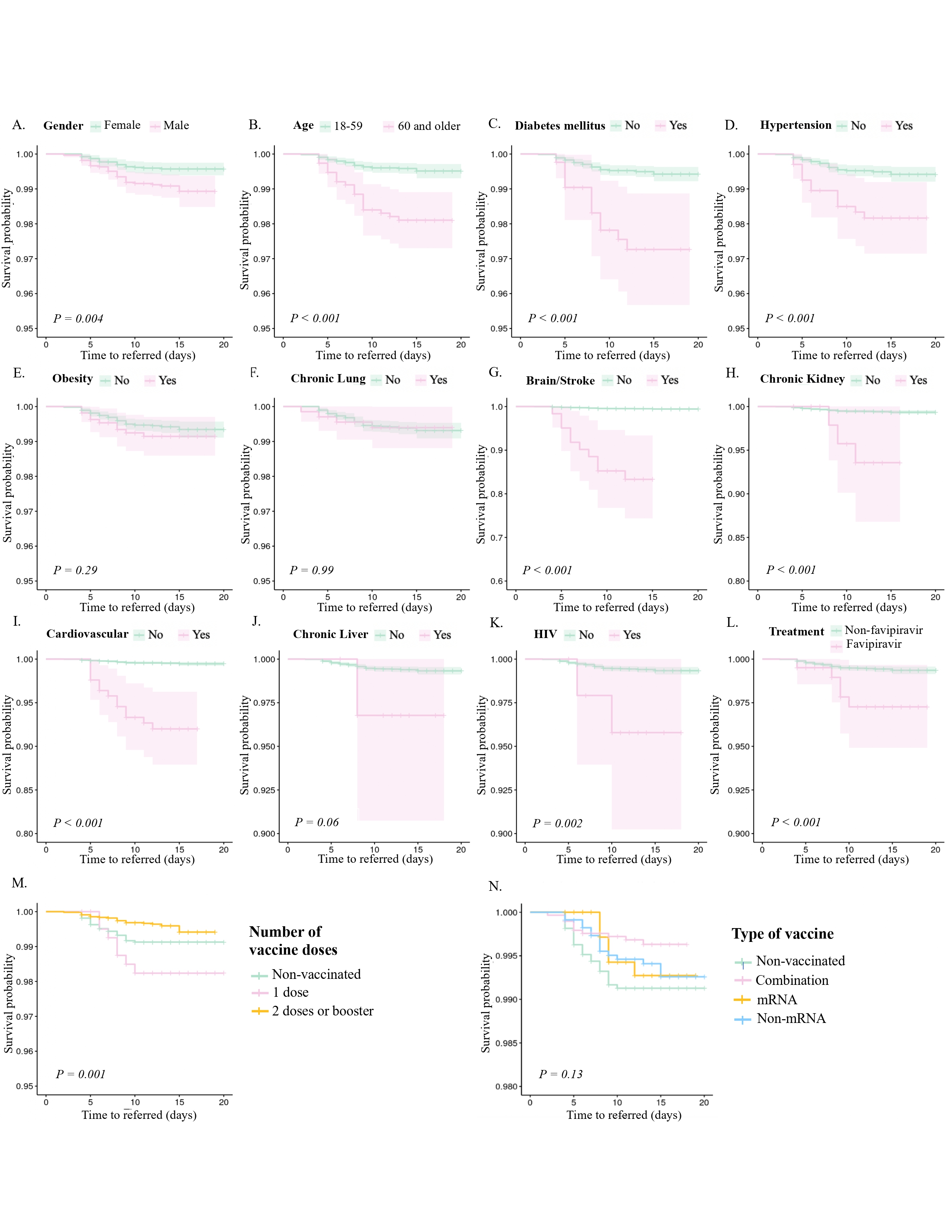

Supplement: Supplementary file 2 [file Image_1.JPEG]
